# Molecular Mechanisms of Immunomodulation Properties of Mesenchymal Stromal Cells: A New Insight into the Role of ICAM-1

**DOI:** 10.1155/2017/6516854

**Published:** 2017-06-27

**Authors:** Yury Rubtsov, Кirill Goryunov, Аndrey Romanov, Yulia Suzdaltseva, George Sharonov, Vsevolod Tkachuk

**Affiliations:** ^1^Department of Medicine, Lomonosov Moscow State University, Lomonosovsky prospect 27-1, Moscow 119192, Russia; ^2^Russian Cardiology Research and Production Complex, 3-rd Cherepkovskaya str. 15-a, Moscow 11552, Russia

## Abstract

Mesenchymal stromal cells (MSC) control excessive inflammation and create a microenvironment for tissue repair protecting from chronic inflammation and tissue fibrosis. We examined the molecular mechanisms of MSC immunomodulatory function in mixed cultures of human adipose-derived MSC with lymphocytes. Our data show that MSC promote unstimulated lymphocyte survival potentially by an increase in antigen presentation. Under inflammatory conditions, mimicked by stimulation of TCR in lymphocytes, MSC suppress activation and proliferation of stimulated T cells. Immunosuppression is accompanied by downregulation of IL-2R*α* that negatively affects the survival of activated T cells. MSC upregulate transcription of indolamine-2,3-dioxygenase (IDO) and inducible NO synthase (iNOS), which generate products negatively affecting T cell function. Both MSC and lymphocytes dramatically increase the surface ICAM-1 level in mixed cultures. Antibody-mediated blockage of surface ICAM-1 partially releases MSC-mediated immune suppression in vitro. Our data suggest that MSC have cell-intrinsic molecular programs depending on the inflammatory microenvironment. We speculate that MSC sense soluble factors and respond by surface ICAM-1 upregulation. ICAM-1 is involved in the control of T cell activation leading to immunosuppression or modest stimulation depending on the T cell status. Immunomodulation by MSC ranging from support of naive T cell survival to immunosuppression of activated T cells may affect the tissue microenvironment protecting from aberrant regeneration.

## 1. Introduction

Mesenchymal stromal cells (MSC) were discovered as fibroblast-like cells from the bone marrow [1]. These cells have mesenchymal surface markers (CD105, CD90, and CD73) and lack hematopoietic surface markers such as CD45 and CD133 [2]. It was shown that MSC are pluripotent and, under certain conditions, can differentiate into chondrocytes, osteocytes, fibroblasts, and adipocytes [3]. Initially, it was thought that the main MSC function is the replacement of dead cells by migration and differentiation in the damage area [4]. But poor survival of transplanted MSC led to revision of their role. Secretion of paracrine factors is currently thought to be the main mechanism of MSC-mediated tissue repair improvement [5]. It is known for certain that MSC support cells that rebuild injured tissue [6] by secretion of soluble angiogenic and neurotrophic factors: vascular endothelial growth factor (VEGF), hepatocyte growth factor (HGF), nerve growth factor (NGF), brain-derived neurotrophic factor (BDNF), and others [7].

During tissue damage, inflammation is a prerequisite condition of productive tissue repair. Cytokines and factors produced in inflamed tissue stimulate migration, proliferation, and differentiation of cells. MSC can possibly protect cells from excessive damage by “controlling” transition from inflammation to repair steps and prevent production of extracellular matrix responsible for fibrosis. It has been shown that MSC possess immunomodulatory activity and are capable of regulating functional activity of lymphocyte and other immune cell types depending on the microenvironment [8, 9]. Activated lymphocytes in vitro secrete soluble factors, such as interferon gamma (IFN-*γ*) and tumor necrosis factor alpha (TNF-*α*) that initiate the MSC immunosuppression program, which induces synthesis of protein factors, in particular, indolamine-2,3-dioxygenase (IDO) and inducible NO synthase (iNOS) [10, 11]. Products of their enzymatic activity (kynurenine and NO) suppress lymphocyte function and proliferation. IDO metabolizes essential amino acid tryptophan and converts it to kynurenine [12]. It is shown that kynurenine and its toxic breakdown products lead to T cell apoptosis [13]. On the other side, the insufficient pool of tryptophan leads to cell cycle arrest in T cells [14].

There is a significant discrepancy concerning realization of MSC immune suppression mechanisms in different species. IDO is one of the key immunosuppression molecules in human MSC, but in mice, there is a dramatic increase in iNOS activity and virtually no changes in IDO level in the course of immunosuppression [15]. The product of iNOS activity is highly reactive nitric oxide (NO) [16] which induces apoptosis of T cells or arrests proliferation [17, 18].

Paracrine mechanisms have a significant part in MSC immunosuppression potential, but MSC possess a much higher suppressive capacity during direct contact with target cells [19]. For example, in case of mice bone marrow-derived MSC incubated with lymphocytes in contact conditions, a tenfold increase in immune suppression activity was shown in comparison to contactless conditions [20]. The elucidation of cell contact-dependent mechanisms of immune suppression is more complicated in comparison to paracrine due to the presence of cell adhesion and costimulation molecules on both MSC's and stimulated immune cell's surfaces [21]. The list of candidate molecules involved in contact mechanisms of immunosuppression was narrowed down to programmed death-1 receptor/programmed death-1 receptor ligand (PD-1/PD-L1) [22], the B7 family immune-regulatory orphan ligand H4 (В7-Н4) [23], intercellular adhesion molecules of adhesion molecule family (ICAM), and vascular cell adhesion molecule (VCAM) [24].

As it was mentioned above, MSC can affect the activity of immune cells and regulate an arresting of inflammatory response [25]. The use of these mechanisms can be considered as an alternative therapeutic tool to treat autoimmune diseases and chronic inflammation [26]. However, recently, it has been demonstrated that MSC, in addition to suppression of immune cells, can stimulate them in the absence of inflammation, regulating immunological status in different conditions [27]. Mechanisms and molecules that trigger this transition are not studied well [28].

In this study, we assessed the influence of MSC (human adipose-derived mesenchymal cells (hASC)) on activated or resting T lymphocytes. Human peripheral blood mononuclear cells (PBMC), containing a significant proportion of circulating T cells, were used as a source of T cells. We established mixed hASC-PBMC cultures under conditions permitting cell contacts or contactless cell cultivation. We found that hASC decreased proliferation of activated lymphocytes by downmodulation of surface IL-2 receptor alpha subunit (CD25) expression. In the case of cell contact-mediated suppression, a significant increase in the ICAM-1 mRNA level in both hASC and T cells was registered, but at the same time, hASC markedly downregulated the ICAM-1 protein level on the T cell surface (similar effects were observed in contactless conditions). Furthermore, ICAM-1 blocking on hASC and the T cell surface significantly restored lymphocyte proliferation and led to upregulation of CD25 surface expression on T cells but did not affect IDO expression, confirming that ICAM-1 acts through a distinct mechanism than paracrine immune suppression. Incubation of hASC with resting lymphocytes augmented their survival and was accompanied by a dramatic increase in the major histocompatibility complex class II (HLA-DR) on the hASC surface, as well as an increase in ICAM-1 on both hASC and T cells.

Our results support the notion that hASC possess both immune stimulatory and immune suppressive properties depending on inflammatory conditions. ICAM can act independently of soluble factors in cell contact-dependent settings of MSC-mediated immune suppression in vitro. It is likely that ICAM-1 serves as molecular switch responsible for activation of the immune suppressive program in hASC.

## 2. Materials and Methods

### 2.1. Materials

The materials used were L-NMMA 1 mМ (Sigma), 1-МТ (Sigma), antihuman ICAM-1 SC-107L (Santa Cruz), antihuman IDO (H-110 sc-25808, Santa Cruz), antihuman iNOS (aa 781-798, Clone 2D2-B2, MAB9502, R&D Systems), antihuman CD28 NA/LE (BD 555725), antihuman CD3 NA/LE (BD 555336), antihuman CD4 (558116, BD PharMingen), antihuman CD25 (555346, BD PharMingen), antihuman CD45 (304026, BioLegend), antihuman CD3 (300431, BioLegend), antihuman CD90 (328110, BioLegend), antihuman CD 73 (344006, BioLegend), antihuman HLA-DR (307610, BioLegend), transwell permeable membranes 0.4 *μ*m (Greiner 665640), TRIzol® Reagent (Invitrogen, 15596-018), CyQUANT®NF (C35006, Invitrogen), Maxima SYBR Green/ROX qPCR Master Mix (2X) (K0221 Thermo Scientific), SuperScript® III Reverse Transcriptase (Thermo Scientific), and SuperSignal™ West Pico Chemiluminescent Substrate (34078 Thermo Scientific).

All donors were notified and agreed to take part in the research according to the draft Declaration on Bioethics and Human Rights (SHS/EST/05/CONF.204/3 REV) paragraph 6 items b and c. All operational procedures and protocols were strictly followed in accordance with MSU bioethics committee guidelines. All experimental protocols were conducted according to the GLP rules signed by the Ministry of Health (Order number 267 from 19 June 2003).

## 3. Methods

### 3.1. Isolation of Adipose-Derived MSC

MSC were isolated from human skin fat by collagenase digestion [29]. Briefly, fat tissue cut in small pieces was incubated with equal volume of collagenase type I/dispase solution (200 u/ml collagenase, Worthington Biochemical M8M10816, 40 u/ml dispase, BD Biosciences 354235 dissolved in DMEM/F12 (HyClone) without supplements) during 30–45 minutes at 37°C. After centrifugation (200*g*) pellet was resuspended in 10 ml of DMEM/F12 supplemented with 100 u/ml penicillin, 100 u/ml streptomycin, 2 mM glutamine, 1 mM sodium pyruvate, and 10% fetal bovine serum (HyClone) (standard culture medium). Suspension was plated at density 50000 tissue fragments/ml, and medium was changed when cells were 70% confluent. After isolation, MSC were analyzed for the CD90, CD73, CD105, and CD45 surface levels.

### 3.2. Lymphocyte Isolation and Activation

25–50 ml of venous blood from healthy individuals was placed into sterile tubes containing EDTA solution (1.5 mg/ml of blood, pH 7.2). Leukocytes were isolated by centrifugation in Ficoll-Paque density gradient (1,077 g/ml, Pharmacia Biotech). Lymphocytes were activated by incubation with phytohemagglutinin solution (10 mkg/ml, Sigma) for 6 hours or plate-bound antibodies against human CD3 (HIT3a 1 ug/ml, BD Biosciences) and human CD28 (CD28.2 0.5 ug/ml, BD Biosciences) for 48 hours in standard culture medium.

### 3.3. Lymphocyte Proliferation Assay

CyQUANT®NF Cell Proliferation Assay Kit (Invitrogen) was used to measure hASC capacity to inhibit activated lymphocyte proliferation. Mixed cultures of hASC and activated lymphocytes were incubated in two different ways: contact (direct interactions between hASC and T cells) and contactless (transwells, cells do not touch each other but can exchange soluble factors), and they were incubated at 37°C and 5% CO_2_ for 48 hours. Lymphocytes were collected by pipetting; cells were pelleted by centrifugation—resuspended in 1X Hanks solution prior to the addition of CyQUANT®NF reagent or permeabilizing solution to determine the basal signal level. Samples were kept in darkness for 1 hour at 37°C. Fluorescence was measured at 530 nm using spectrophotometer Zenyth 3100 (Anthos). Unstimulated lymphocytes were used to plot the calibration curve.

### 3.4. Immunophenotyping and FACS

Cells were harvested, washed twice in PBS, and resuspended in 100 ml of PBS with 1% BSA (Sigma). Cell suspensions of hASC or lymphocytes were incubated with fluorophore-conjugated antibodies against surface markers according to manufacturer's instructions. Cells were collected, centrifuged, washed in PBS/BSA, and analyzed by FACS using FACS Canto II (BD Bioscience) instrument. Data were analyzed using FlowJo software.

### 3.5. Inhibitory Analysis

Kynurenine competitive ELISA kit (BA E-2200 LDN) was used to measure the kynurenine level. Culture supernatants incubated with 1 mM IDO inhibitor, 1-methyl-DL-tryptophan, or solvent control (DMSO) were transferred into a 96-well plate with adsorbed acylated competing antigen. Levels of kynurenine were calculated by measuring absorbance at 405 nm and comparing absorbance values with the reference curve plotted using standard solution provided by the manufacturer. All results were normalized on values of the kynurenine level in supernatants of hASC cultures.

To measure the NO level, we used Nitric Oxide Colorimetric Assay Kit (K262-200, Biovision). Supernatants of mixed cultures incubated with or without 1 mM iNOS inhibitor NG-monomethyl-L-arginine were incubated with nitrate reductase to convert all nitrates to nitrite. After incubation with Griess reagents, absorbance was measured at 540 nm. Absorbance values were normalized to control values obtained from hASC supernatants.

### 3.6. RT-PCR

Cells were dissolved in 800 ul of TRIzol (Invitrogen) and incubated overnight at 4°C prior to standard isolation of RNA. RNA samples treated with DNAse I (EN0521 Thermo Scientific, 37°C, 20 min) were used for reverse transcription by SuperScript III (Invitrogen). RT-PCR was conducted using 2X Maxima SYBR Green/ROX qPCR Master Mix (Thermo Scientific), containing 10 mM dNTPs with dUTP, AmpliTaq Gold® DNA Polymerase (5000 u/ml), SYBR Green I dye, and passive internal reference 1. PCR product accumulation was registered using DTprime Real-time Detection Thermal Cycler DT96 (DNA technology). PCR primer sequences are listed in Supplementary Material available online at https://doi.org/10.1155/2017/6516854.

### 3.7. Western Blotting

Protein extracts were separated by SDS-PAGE and transferred to nitrocellulose membrane (Hybond-C Extra, GE Healthcare) using Mini Trans-Blot® Cell system (Bio-Rad) according to manufacturer's instructions. Membrane was incubated with primary antibodies against IDO 1 : 700 (sc25808), iNOS 1 : 500 (aa 781-798, Clone 2D2-B2, MAB9502, R&D Systems). After washing, membranes were incubated with the corresponding secondary antibodies. Signals were normalized against vinculin (CST polyclonal #4650) for IDO, against GAPDH (CST 14C10 #2118) in case of iNOS. For iNOS, control lysates of human umbilical vein endothelial cells (hUVEC) incubated with 25 ng/ml HGF or VEGF for 20 hours were used. Secondary antibodies were kindly gifted by Dr. Francesco Blasi (IFOM-IEO). Protein bands were visualized using West Pico Chemiluminescent Substrate (Thermo Scientific).

### 3.8. Data Analysis

Data analysis was performed using Prism GraphPad 5.0 and Statsoft Statistica 6.0. Data was represented by mean ± standard error. For normality analysis, Kolmogorov-Smirnov test was performed. For nonparametric data, Kruskal-Wallis ANOVA ranked test was conducted. For paired analysis (activated, not activated lymphocytes), Mann-Whitney *U* test was conducted. ^∗^*р* < 0.05, ^∗∗^*p* < 0.01, and ^∗∗∗^*p* < 0.001.

## 4. Results

### 4.1. hASC Suppress PBMC Proliferation in Mixed Cultures

**Figure 1:**
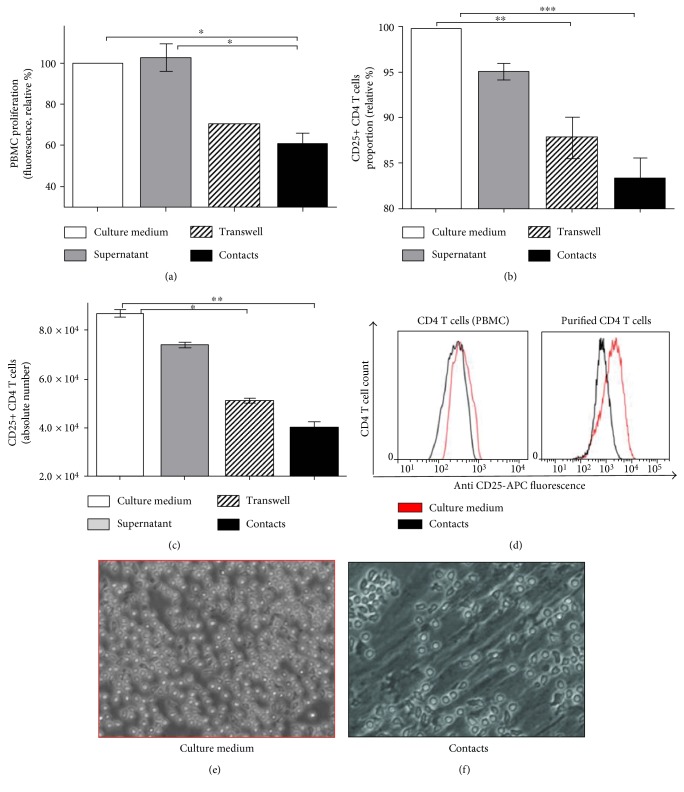
hASC suppress lymphocyte proliferation and downregulate IL-2R*α* on activated T cells. Proliferation of PBMC activated with PHA and cultured separately and with hASC in contact or contactless conditions (25 : 1 ratio, 48 h) (a). CD25 expression in PBMC stimulated with plate-bound anti-CD3 and anti-CD28 and cultured with hASC (25 : 1, 48 h, mean + SEM, *n* = 5). Culture medium and medium conditioned by hASC served as negative controls. Proportion and absolute number of CD25+ CD4 T cells determined using immunofluorescent staining and FACS analysis are shown (mean + SEM, *n* = 6) (b, c). Similar analysis was performed using FACS sorted from PBMC pure (>95%) human CD4 T cells incubated with hASC (right plot); results obtained using total PBMC stained for CD25 are shown on the left plot; data of representative experiments are shown as histograms (d). Representative images of live PBMC-hASC cultures acquired using light microscope equipped with a digital camera. (48 h of incubation, magnification—10х) (e, f) (^∗^*p* < 0.05, ^∗∗^*p* < 0.01, and ^∗∗∗^*p* < 0.001).

To determine hASC immune suppressive potential in vitro, we established an experimental cell-based in vitro suppression assay. hASC and PBMC were isolated from fat tissue and venous blood of healthy donors (*n* = 6 and *n* = 4, resp.). Donor hASC were cultured with activated T cells, which were isolated as a part of donor PBMC preparation (PBMC typically contain approximately 70% of T cells) [30]. To activate T cells, we used either phytohemagglutinin (PHA) or plate-bound anti-CD3 and anti-CD28 antibodies. We cultured hASC with activated PBMC by contact and contactless methods. Transwell membranes permeable to soluble factors but impermeable to cells were used to separate PBMC and hASC. Using this approach, we have found that lymphocyte proliferation inhibition was the highest after 48 hours of culturing. By using different hASC to PBMC ratios, we observed that hASC-mediated suppression is cell number dependent and shows the best effect (optimal for T cell suppression) at hASC:PBMC cell ratio 1 : 25 in contact settings (Figure 1(a)). To make sure that lymphocytes harvested for proliferation assay are not contaminated with hASC, we stained PBMC samples with antibodies against MSC's surface marker CD73 (Figure S1); only about 1% of cells carried CD73 and were negative for CD45. By using FACS-sorted CD4 T cells, we confirmed that similar effects could be detected using purified CD4 T cell population in suppression assay (Figure S2(a)). Unstimulated PBMC, containing resting T cells and growth medium conditioned by hASC, were used as a negative control. Stimulated PBMC cultured alone were used as a positive control taken for 100% proliferation.

### 4.2. hASC Suppress CD4 T Cell Activation by Limiting IL-2R*α* (CD25) Expression on the Surface of Activated Lymphocytes

The lymphocyte number decrease in mixed cultures with hASC can be potentially caused by two major reasons: lymphocytes can become more sensitive to apoptosis and die at a faster rate or they can become less proliferative due to phenotypic changes that render their sensitivity to IL-2 and other proinflammatory cytokines. Sensitivity of T cells to IL-2 is directly linked to the surface level of IL-2R*α* (CD25) subunit which is necessary to form high-affinity IL-2 receptor on the lymphocyte surface. High-affinity IL-2 receptor provides the signal required to sustain T cell proliferation [31]. We evaluated the surface level of CD25 in stimulated lymphocytes in in vitro suppression assay using fluorophore-conjugated anti-CD25 antibody staining followed by FACS analysis. We detected a statistically significant decrease in the proportion and number of CD4 T cells with a high level of CD25 in samples where stimulated T cells were incubated with hASC in comparison to control samples (Figures 1(b) and 1(c)). We observed this effect both in cell contact and contactless conditions, but immunosuppression was significantly higher under cell contact conditions (Figures 1(d)–1(f)).

To assess changes in T cell apoptosis, we stained T cells harvested from hASC-PBMC cultures with fluorophore-conjugated annexin V. This staining detects phosphatidylserine exposed on the cell surface during early stages of apoptosis and hence discriminates viable cells from apoptotic. Analysis of the proportion of apoptotic cells did not reveal any statistically significant changes in samples with marked immune suppression in comparison to those in control samples (see supplementary data). Our data suggest that CD25 downmodulation on the surface of activated T cells is likely responsible for in vitro immunosuppression effect in our model system.

### 4.3. IDO Secretion Is a Main Mechanism of Contact-Independent Immune Suppression

**Figure 2:**
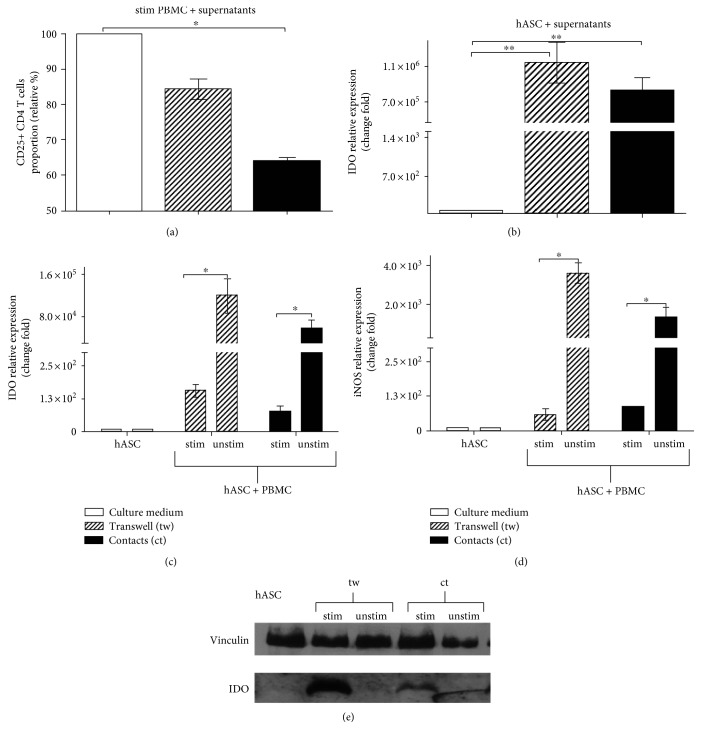
Increase in IDO level in hASC in the presence of activated PBMC. Supernatants from mixed cultures of activated PBMC (anti-CD3, anti-CD28) cultured with hASC (48 h) affect the CD25 level in CD4 T cells. After 48 hours of incubation, PBMC were analyzed for CD25 (IL-2R*α*) surface expression (*n* = 3, mean + SEM) (a). Changes in IDO transcription after treatment of hASC with supernantants (*n* = 3) (b). Transcriptional activity of IDO (c) and iNOS (d) genes in the course of hASC-mediated immune suppression (*n* = 4). IDO protein level in hASC cultured with anti-CD3- and anti-CD28-stimulated PBMC at 1 : 25 ratio for 48 hours (vinculin-normalization control) (e) (^∗^*p* < 0.05 and ^∗∗^*p* < 0.01; unstim—unstimulated PBMC, stim—PBMC stimulated with anti-CD3 and anti-CD28).

hASC use paracrine mechanisms to suppress T cell function. To determine the impact of soluble factors on hASC-mediated suppression of T cells, we added growth medium conditioned by hASC cultured with T cells (in contact and contactless conditions) and hASC cultured alone to both stimulated PBMC and hASC, respectively. We expected to assess a decrease in T cell proliferation associated with downmodulation of the surface IL-2R*α* (CD25) level in T cells in the case of supernatants of cultures where we detected marked immune suppression by hASC. We thought to detect elevated production of effector molecules responsible for immune suppression in hASC, in particular, IDO and iNOS. Indeed, we determined that the CD25 level drops in lymphocytes incubated with medium collected from mixed hASC-activated T cell cultures in comparison to hASC supernatants. Again, conditioned medium from hASC-T cell cultures permitting contacts between two cell types had more profound suppressive effects on T cells in comparison to those in samples, where supernatant from contactless cultures of hASC with activated T cells was used (Figure 2(a)). We also documented a significant increase in the transcription rate of IDO and iNOS genes according to the assessment of the mRNA level in samples containing hASC treated with respective supernatants (Figures 2(b)). Transcriptional activation of IDO and iNOS genes was higher in contrast to changes in CD25 expression in mixed hASC-PBMC cultures when T cells and hASC were separated than in contact conditions (Figures 2(c) and 2(d)). To prove that transcriptional activation results in elevated synthesis of respective proteins, we measured IDO and iNOS protein levels in hASC lysates. The IDO protein level was significantly higher in hASC cultured in contact with activated T cells in comparison to hASC incubated with resting T cells. An increase in the IDO level was even higher in hASC incubated with activated T cells in contactless conditions in accordance with data published earlier [11] (Figure 2(e)). We failed to detect iNOS by western blot analysis in hASC lysates, used also for IDO analysis (data are not shown), despite a strong increase in iNOS gene transcription.

**Figure 3:**
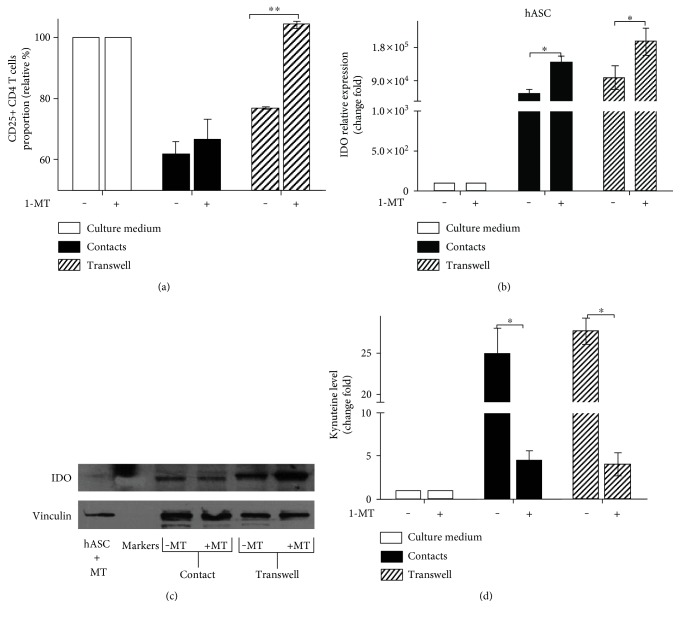
IDO inhibition blocks paracrine hASC-mediated immune suppression. Activated PBMC were cultured with or without hASC in contact and contactless conditions in the presence of 1-MT for 48 hours. Proportion of CD25+ CD4 T cells (a); IDO mRNA changes in hASC (b); IDO protein level in hASC cell lysates (c); kynurenine level in supernatants from hASC-PBMC cultures. Absorbance values (405 nm) were normalized to control samples (supernatant from hASC cultured alone). (d) All data represent mean + SEM of three independent experiments (1-MT—1-methyl-DL-tryptophan; ^∗^*p* < 0.05 and ^∗∗^*p* < 0.01).

To confirm IDO involvement in hASC-mediated immune suppression, we used the specific inhibitor of IDO enzymatic activity 1-methyl-DL-tryptophan. An addition of 1-MT to cultures in in vitro suppression assay partially released proliferative block increasing CD25+ CD4 T cells proportion in contactless conditions and to a lesser extent in contact (Figure 3(a)). At the same time, in the presence of an inhibitor, we registered a marked increase in IDO transcription in hASC incubated with activated lymphocytes in contactless conditions (Figure 3(b)). The elevated level of IDO transcript and its respective protein product (Figure 3(c)) correlated with lower kynurenine levels in the medium (Figure 3(d)). This result shows that the inhibitor affects IDO enzymatic activity and, probably, induces compensatory boost of IDO transcription. These observations reveal that IDO enzymatic activity is critical for hASC-mediated immune suppression especially in contactless conditions [32].

### 4.4. hASC Support Survival of Resting Lymphocytes

**Figure 4:**
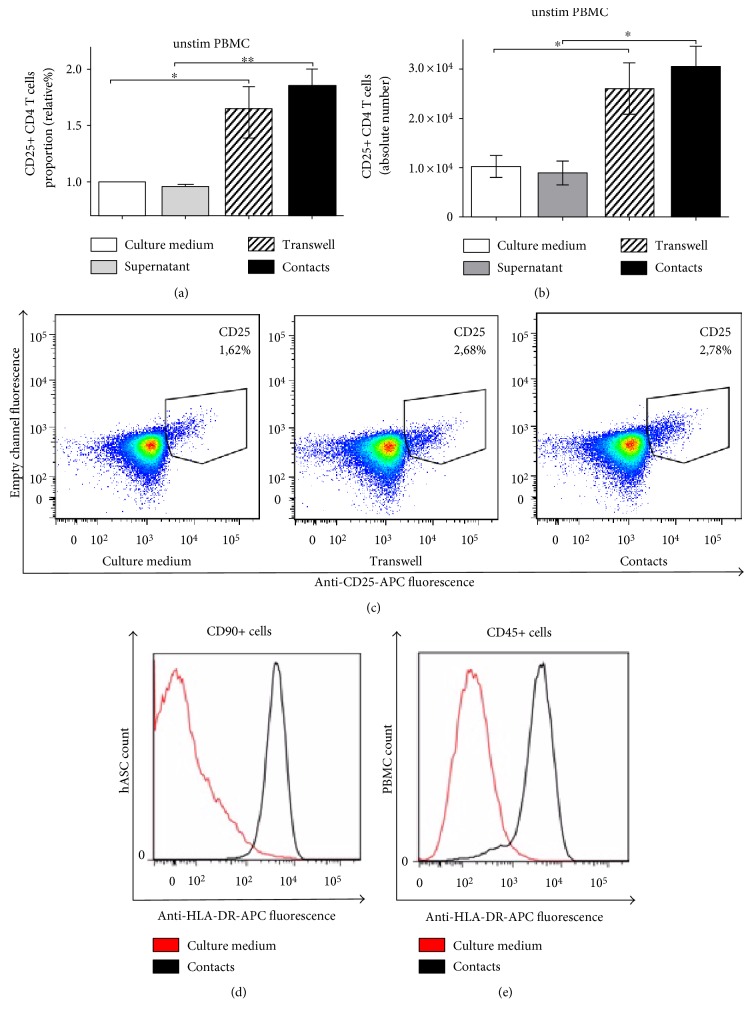
hASC support survival of unstimulated T cells. CD25 expression in unstimulated PBMC cultured with hASC in contact and contactless conditions (48 h). Proportion (a) and absolute number (b) of CD25+ CD4 T cells (*n* = 6). Representative dot plot of CD25 expression on unstimulated T cells cultured with hASC (c). HLA-DR expression in nonstimulated PBMC cultured alone or with hASC under different conditions for 7 days. hASC (d) and PBMC (e), respectively (^∗^*p* < 0.05 and ^∗∗^*p* < 0.01).

hASC can suppress activated T cells; however, there are controversial reports claiming that in vitro MSC can support survival and to some extent activate resting T cells [26, 27]. We tested the hASC ability to affect survival and activation of resting T cells in mixed cultures. We detected an increase in lymphocyte numbers after 48 hours of incubation with hASC. Increase in cellularity was accompanied by the elevated level of CD25 on the surface of CD4 T cells (Figures 4(a)–4(c)). We further documented that hASC support the survival of purified CD4 T cells (Figure S2(b)). This finding suggests that hASC in the absence of antigenic stimulation can provide survival and/or activation signals to resting T cells. To detect these signals, we focused on phenotypic changes in hASC following incubation with T cells. We detected significant upregulation of major histocompatibility complex (MHC) class II on hASC surface expression after incubation with PBMC (Figure 4(d)). This result suggests that in the absence of inflammation, MSC can potentially serve as antigen-presenting cells, providing a tonic signal to resting T cells by presenting low-affinity self-antigens. Unexpectedly, we detected a dramatic increase in HLA-DR (MHC II) surface expression in a fraction of PBMC after incubation with hASC (Figure 4(e)). This finding provides evidence that in vitro hASC positively affect antigen-presenting properties of immature antigen-presenting cells (monocytes), comprising PBMC, by increasing their HLA-DR expression.

### 4.5. ICAM-1 Upregulation in hASC and Lymphocytes

**Figure 5:**
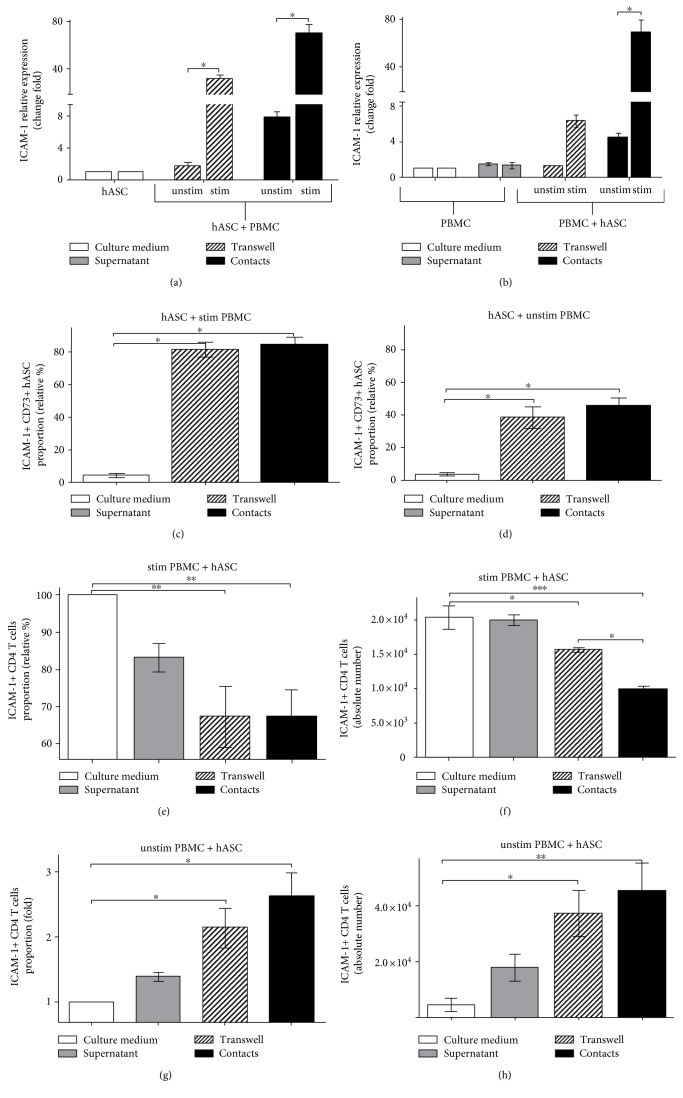
ICAM-1 level changes in the course of hASC-mediated immunosuppression. ICAM-1 mRNA levels in hASC (a) and PBMC (b) from mixed cultures. ICAM-1 surface expression on hASC (c, d) and stimulated (e, f) or unstimulated (g, h) PBMC from mixed cultures (all data represent mean + SEM, *n* = 6, ^∗^*p* < 0.05, ^∗∗^*p* < 0.01, and ^∗∗∗^*p* < 0.001).

We compared hASC-mediated T cell suppression under cell contact and contactless conditions and detected a distinction between these two types of hASC-T cell interactions. For instance, we observed a more profound proliferative arrest in the case of contact hASC-T cell cultures in comparison to contactless samples. At the same time, we recorded higher IDO mRNA and protein levels in the case of contactless soluble factor-dependent immune suppression. This fact implies that aside from previously described paracrine factors (IDO, iNOS, and certain cytokines), hASC carry on the cell surface factors playing a role in contact-dependent immune suppression. Results of in vivo experiments in mice suggest that it could be cell adhesion molecules, such as ICAM or VCAM [24]. To narrow the list of potential molecules involved in contact hASC-mediated immune suppression, we analyzed changes in transcriptional activity of a set of cell adhesion protein genes in hASC and T cells (PBMC) in the course of in vitro immune suppression. We found that in both hASC (Figure 5(a)) and PBMC (Figure 5(b)), only ICAM-1 mRNA dramatically increased in mixed cultures of hASC and activated PBMC. Moreover, we detected ICAM-1 transcriptional activation even when hASC were incubated with unstimulated PBMC (Figures 5(a) and 5(b)).

To prove that upregulation of ICAM-1 gene transcription results with increase in the protein level, we assessed ICAM-1 on the surface of hASC and T cells. Using FACS analysis of cells stained with anti-ICAM-1 mAb, we documented that in the presence of both activated and unstimulated PBMC, hASC upregulate ICAM surface expression (Figures 5(c), 5(d), and Figure S3).

### 4.6. ICAM-1 Level on the Surface of Activated Lymphocytes Decreases in the Presence of hASC

We suggested that decrease in the IL-2R*α* (CD25) level on activated lymphocytes could probably correlate with ICAM-1 level changes on the cell surface. Using ICAM surface staining, we determined that the decrease in the ICAM-1 level correlated with downmodulation of IL-2R*α* on activated lymphocytes in the course of hASC-mediated immune suppression (Figure 5(e)). Moreover, we also registered the decrease in the absolute number of T cells expressing the high level of ICAM-1 on the surface (Figure 5(f)).

### 4.7. hASC Induce Surface Expression of ICAM-1 in Resting T Cells

Changes of the ICAM-1 level on activated lymphocytes suggest that ICAM-1 could be used as a marker of lymphocyte activation. Therefore, we proposed that hASC should affect the ICAM-1 level on the surface of unstimulated lymphocytes (PBMC) in contact conditions. We measured surface ICAM-1 dynamics on the T cell surface in mixed cultures of hASC with unstimulated PBMC and found a slight increase in the ICAM-1 level in contactless conditions in comparison to PBMC cultured alone. Augmentation of ICAM-1 in cell contact conditions was much higher than in contactless (Figure 5(g)). Numbers of ICAM-expressing cells were also higher in unstimulated (PBMC) cultured with hASC in contact conditions. (Figure 5(h)). These data support the hypothesis that hASC affect the ICAM-1 level on T cells depending on the following conditions: they negatively regulate ICAM-1 in activated T cells and upregulate ICAM-1 in resting T cells.

### 4.8. Soluble Factors Regulate ICAM-1 Level on the Surface of Both hASC and Lymphocytes

**Figure 6:**
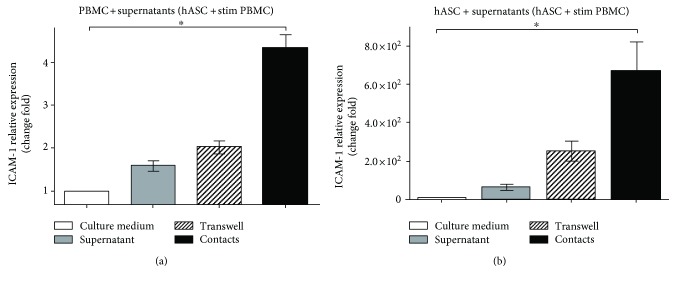
Supernatants from mixed cultures of hASC and activated PBMC stimulate ICAM-1 transcription in hASC and PBMC. PBMCs were activated with anti-CD3 and СD28 and cultured alone or with hASC in contact and contactless conditions (48 h). Supernatants were added to activated PBMC and hASC cultured alone. PBMC (a) and hASC (b) were analyzed for ICAM-1 mRNA level 48 hours later (mean + SEM, *n* = 3, ^∗^*p* < 0.05).

We suggested that, apart from contact interactions, paracrine mechanisms may also contribute to ICAM-1 regulation in hASC and lymphocytes. To test our hypothesis, we added supernatants collected from cultures of hASC with activated lymphocytes to both activated PBMC and hASC cultured alone. After two days of incubation, hASC and T cells were assessed for changes in ICAM-1 gene expression. We determined that the transcriptional activity of ICAM-1 gene in both hASC and PBMC was augmented in the presence of supernatants. Thus, we documented that adding supernatants containing soluble factors leads to elevated ICAM-1 gene transcription in both lymphocytes (Figure 6(a)) and hASC (Figure 6(b)), suggesting that soluble factors affect ICAM-1 transcriptional regulation. It is noteworthy that supernatants collected from cultures, where hASC-contacted T cells had stronger effect on ICAM-1 transcription level than the ones collected from contactless cultures.

### 4.9. ICAM-1 Blocking Releases hASC-Mediated Immune Suppression and Restores the CD25 Level on the Surface of Activated CD4 T Cells Independently of IDO

**Figure 7:**
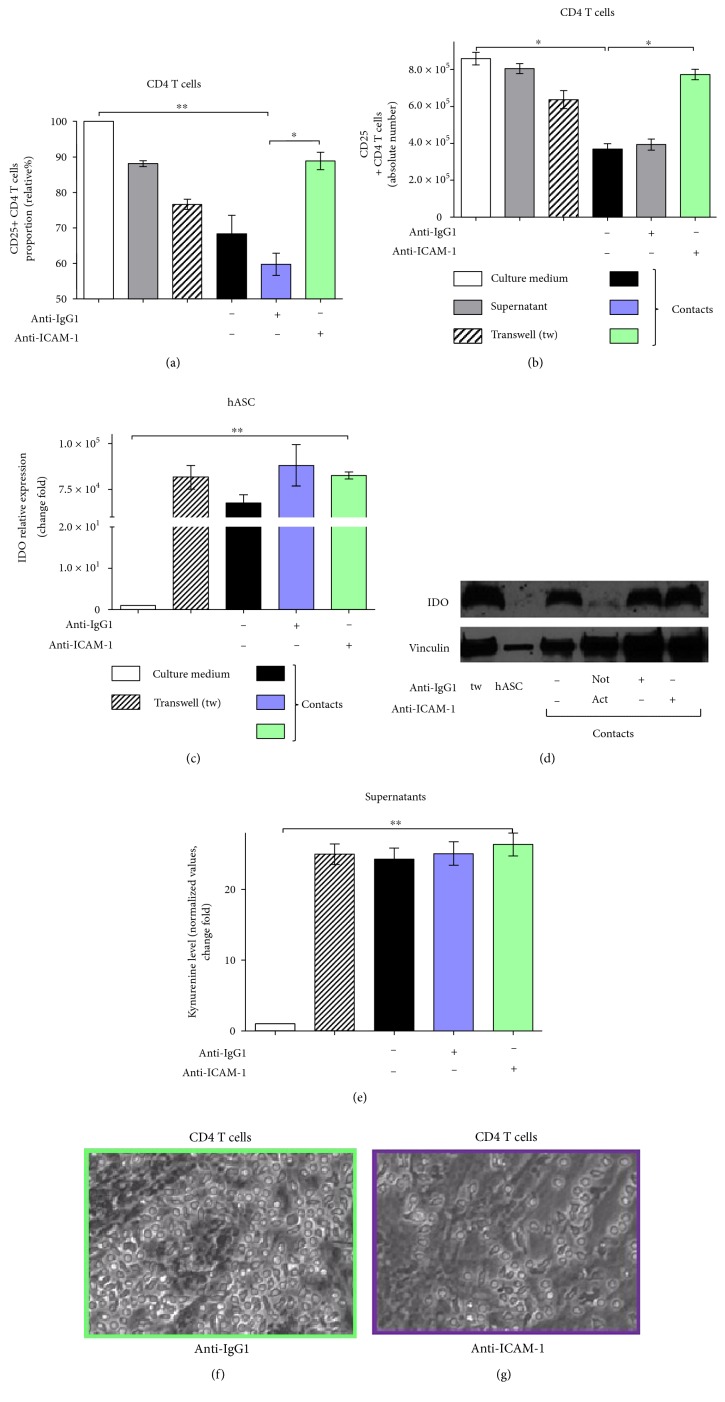
ICAM-1 blocking inhibits hASC-mediated immunosuppression independently of IDO. CD3+ CD4 T cells sorted from PBMC and activated with anti-CD3/anti-СD28 were cultured alone or with hASC in the presence of anti-ICAM-1 or control IgG1 in contact or contactless conditions for 48 hours. Proportion and absolute numbers of CD25+ CD4 T cells were determined (a, b, resp.). hASC IDO gene expression (c); IDO protein level (d). Kynurenine level in mixed cultures (e) (mean + SEM, *n* = 6, ^∗^*p* < 0.05 and ^∗∗^*p* < 0.01). T cell-hASC cultures after control IgG1 (e) or anti-ICAM (f) treatment. Magnification 10x.

ICAM-1 downregulation in activated lymphocytes, caused by contacts with hASC, suggests that ICAM-1 could possibly participate in cell contact-dependent immune suppression by hASC. To test that, we blocked ICAM-1 on the surface of hASC and T cells using ICAM-specific antibodies and analyzed an effect of ICAM blockage on hASC-mediated immune suppression. We determined that incubation with ICAM-1-blocking antibodies leads to a significant increase in CD25 on the surface of lymphocytes (Figures 7(a), 7(b), and Figure S4) in comparison to samples with control IgG1 in cultures of activated T cells with hASC. These data can be considered as evidence that ICAM-1 is directly involved in immune suppression by hASC in vitro. To test if ICAM-1 can affect the IDO level and indirectly regulate immune suppression, we assessed IDO transcription and protein levels in the conditions of ICAM-1 antibody blockage. Surprisingly, antibody-mediated ICAM-1 blockage did not affect the IDO level on both transcriptional and protein levels (Figures 7(c) and 7(d)). We also detected no changes in the kynurenine level (Figure 7(e)). This finding points to nonoverlapping roles for ICAM-1 and IDO in hASC-mediated immune suppression. Additional experiments are needed to further elucidate the detailed molecular mechanism of ICAM involvement in contacts between MSC and T cells.

## 5. Discussion

It is well known that MSC possess immunomodulatory activity both in vivo and in vitro [25]. Molecular mechanisms of MSC-mediated immune modulation have been extensively studied. It has been shown that proinflammatory cytokines trigger receptors on the surface of MSC leading to activation of genes and increase in a level of the so-called effector molecules, such as IDO and iNOS, and secretion of immune suppressive cytokines, in particular, transforming growth factor beta (TGF-*β*) and IL-10 [20, 33]. This set of molecules negatively affects activated T cells and dendritic cells and possibly stimulates conversion of conventional effector CD4 T cells to regulatory T cells (Treg), specific T cell lineage possessing strong immune suppressive properties [34]. Direct restriction of T cell activation, limitation of DC maturation, and hence antigen presentations, as well as generation of Treg cells, serve as major mechanisms of MSC-mediated immune suppression directed mainly towards activated T cells [35].

Despite understanding of the “big picture”, a significant controversy concerning the difference in molecular mechanisms between cell contact-independent paracrine and cell contact-dependent immune suppression exists [36]. According to the modern paradigm, contact-independent immune suppression is mainly driven by the increase in amount and activity of IDO in MSC, the enzyme converting tryptophan to kynurenine, which limits activation and proliferation of T cells [14]. On the other side, contact-dependent MSC-mediated immune suppression mostly relies on increase and activity of iNOS [17]. Participation of other soluble factors, such as IL-10, TGF-*β*, and PGE2 is not excluded completely but has limited impact and is rather controversial [26, 37]. Recent studies demonstrate that the program of immune suppression by MSC is not a hard-wired mechanism but, instead, has a significant degree of plasticity [38]. It has been shown that a significant discrepancy between mechanisms of mouse and human MSC-mediated suppression exists. Murine MSC mainly use iNOS, while human cells IDO [10, 15] which suggests that results of MSC studies in murine models should be verified in human cells.

We demonstrated that hASC-mediated immune suppression in both cell contact and cell contactless conditions is associated with a marked increase in transcription and accumulation of IDO protein in human MSC isolated from skin fat (Figures 2(d) and 2(e)). Upregulation of IDO is associated with an increase in the kynurenine level in supernatants (Figure 3(d)). Despite significant upregulation of iNOS gene transcription (Figure 2(d)), the iNOS protein level in hASC (according to western blot Figure S5(a)) and NO level do not change in culture medium in mixed PBMC-hASC cultures; thus, there is no change in CD25+ CD4 T cells proportion. (Figures S5(b) and S5(c)). These data support the notion that IDO in human cells can serve as the main executor of MSC-mediated immune suppression. Assessment of activation status of T cells cultured with hASC allowed us to reveal that immune suppression leads to a decrease in CD25 expression on the surface of T cells (Figures 1(b)–1(d)) and does not affect the proportion of apoptotic T cells (data are not shown) in accordance with recent data [19, 20, 39]. CD25 is required to maintain T cell proliferation and survival [31]. We can only guess if the decrease in CD25 expression occurs prior to the decrease in T cell activation or is a reason of it. But there is a correlation between CD25 downmodulation and hASC-mediated immune suppression of T cells (Figures 1(a) and 1(b)). We failed to detect the marked changes in Foxp3 expression in CD4 T cells in hASC-PBMC (T cell) cultures under the most tested conditions (Figures S6(a) and S6(b)). This result suggests that immunomodulatory effects observed in our study cannot be explained by changes in Treg population. Failure to detect changes in the proportion of Foxp3+ T cells could be caused by lack of constant TCR stimulation in our mixed cultures. Also, we evaluated Treg proportion at early time points (48 hours) in our study in comparison to that in other studies [40], while accumulation or loss of Treg may take more time.

Unexpectedly, we have found that blockade of surface ICAM-1 in contact hASC-PBMC cultures results in dramatic loss of Foxp3-expressing CD4 T cells (Figure S6(a)). Observed disappearance of Treg, which normally suppress T cell proliferation and activation, could potentially explain augmented proliferation of T cells caused by blockage of ICAM. On the other side, ICAM expression is required for T cell activation and ICAM blockage should result in opposite effect on in vitro stimulated T cells. Additional studies are needed to figure out if ICAM-1 blocking directly affects expression of Foxp3 by Treg or triggers another mechanisms involved in the survival of Treg.

Another important issue is the difference in mechanisms of cell contact-dependent and cell contact-independent MSC-mediated immune suppression. We and others demonstrated that cell contact immune suppression is more potent than contactless [17, 20]. It is based on upregulation of cell adhesion molecules, namely, ICAM-1 and VCAM-1, on the surface of murine MSC in the course of immune suppression. ICAM-deficient murine MSC have a defect in immune suppression in vivo, and in vitro blockade of ICAM and VCAM on the surface of MSC results in partial release of immune suppression [24]. We found that in comparison to murine MSC, ICAM is the only cell adhesion molecule, which expression significantly increases during incubation of hASC with PBMC (or sorted human CD4 T cells) (Figure 5(a)). Our data suggest that soluble factors could be involved, since we detected an increase in the ICAM-1 mRNA level, following the addition of supernatants from contact and contactless hASC-PBMC cultures to hASC and activated lymphocytes cultured separately (Figures 6(a) and 6(b)). According to previous reports, expression of adhesion molecules on MSC can be induced by incubation with cytokines and depends on cytokine signaling, at least in murine MSC [24]. At the same time, expression of IDO can be induced in murine and human MSC by incubation with high concentrations of proinflammatory cytokines, such as IFN-*γ*, TNF-*α*, IL-1, and their combinations [10, 34]. This suggests that cell adhesion-induced signaling and IDO expression could be linked. But in our hands, ICAM-1 upregulation in hASC does not depend on T cell activation and correlates with hASC capacity to inhibit activation of T cells and to decrease ICAM-1 expression on the surface of activated T cells (Figures 5(e) and 5(f)). ICAM blockage did not affect IDO level (Figures 7(c)–7(e)) suggesting that ICAM-mediated binding of hASC with T cells does not affect signaling involved in IDO production. We did not detect dramatic changes in IFN-*γ* or TNF-*α* concentration in hASC-PBMC cultures used in our study (we measured levels of cytokines and chemokines in supernatants from hASC-PBMC cultures, data are not shown). We assume that changes in other secreted or surface molecules or combination of multiple factors could be required to regulate ICAM expression in hASC. Antibody-mediated blockade of ICAM-1 on both hASC and T cells led to significant release of hASC-mediated immune suppression and upregulation of the CD25 surface level in T cells (Figures 7(a) and 7(b)). These data support the idea that ICAM-1-mediated binding between hASC and T cells may be responsible for providing additional signals required for immune suppression and probably does not influence secretion of soluble factors involved in immune modulation.

We cannot rule out the possible role of VCAM-1 in hASC-PBMC interactions, since Majumdar et al. have shown that there is strong adhesion between MSC from the human bone marrow and activated T cells dependent on VCAM-1-*α*4 integrin interactions [41]. But we and others confirmed [24] that VCAM expression can be induced only under MSC exposure to high TNF-*α* concentrations (20 ng/ml or higher). Thus, we speculate that VCAM-1 can play a “back-up” role in hASC contact-dependent immune suppression.

We also examined mechanisms of immune stimulation by MSC. We assessed activation status and survival of resting T cells from PBMC cultured with hASC. A statistically significant increase in the CD25 surface level indicated that in the presence of hASC, T cells were more “activated” (Figures 4(a)–4(c)). We also found that purified T cells survive better in the presence of hASC (Figure S1(b)). Moreover, we documented that ICAM-1 blockage reverses hASC-mediated immune stimulation of resting purified T cells or PBMC in mixed cultures (Figures S7(a), S7(b), and Figure S8). The positive effect of MSC on resting T cells may be explained by dramatic upregulation of the surface level of class II histocompatability molecules (HLA-DR) on the surface of hASC cultured with purified resting CD4 T cells in contact conditions (Figure 4(d)). We also found that a fraction of PBMC also significantly increase the HLA-DR level on the surface (Figure 4(e)) following incubation with hASC. These data suggest that factors secreted by hASC and the increase in surface HLA-DR on myeloid cells in PBMC may be responsible for tonic stimulation and survival signals, promoting survival of resting T cells.

Taken together, our data provide insights of molecular mechanisms of human MSC-mediated immune suppression. We show that MSC possess immune suppressive or immune stimulatory properties in mixed cultures with T cells depending on activation status of immune cells. ICAM-1 in human MSC is a molecule involved in contact immune suppression in vitro and potential switching between contactless and contact immune suppression. These data suggest that MSC can be suitable for clinical use not only for treatment of inflammatory and autoimmune diseases but also for transplantation to support tissue remodeling and regeneration.

## Supplementary Material

Supplementary material contains experimental data concerning hASC immunosuppression and their impact on target lymphocytes. We assessed proportion of proliferating lymphocytes in mixed cultures with hASC using CyQuant method. To be sure that lymphocyte samples are not contaminated with hASC we used FACS analysis of cell suspensions stained with antibodies against hASC surface marker CD73. By using this approach we demonstrate that contamination of lymphocytes with hASC is negligible (Figure 1). To confirm that effects of hASC on PBMC are mostly defined by hASC influence on T cells, we set mixed cultures of hASC with CD4 T cells FACS-sorted from human PBMC. Results of these experiments demonstrate that hASC preferentially act on T cells in PBMC (Figure 2). Figures 3, 4 and 8 contain data obtained using FASC analysis of surface markers in the course of immune suppression experiments under conditions of ICAM surface blockage and control experiments. Figure 5 provides data suggesting that changes in iNOS mRNA level in hASC during immune suppression are not accompanied by changes in protein level and enzymatic activity according to NO level measurements in culture media. Figure 6 demonstrates that ICAM antibody blockage somehow inhibits survival and/or proliferation of CD4 T regulatory cells with phenotype CD4CD25Foxp3. Figures 7 and 8 show that hASC can support survival of resting T cells in mixed cultures.
